# Pimozide Suppresses the Growth of Brain Tumors by Targeting STAT3-Mediated Autophagy

**DOI:** 10.3390/cells9092141

**Published:** 2020-09-22

**Authors:** Alok Ranjan, Itishree Kaushik, Sanjay K. Srivastava

**Affiliations:** 1 Department of Biomedical Science, Texas Tech University Health Sciences Center, Amarillo, TX 79106, USA; alok.ranjan@ttuhsc.edu (A.R.); i.kaushik@ttuhsc.edu (I.K.); 2 Department of Immunotherapeutics and Biotechnology, Texas Tech University Health Sciences Center, Center for Tumor Immunology and Targeted Cancer Therapy, Abilene, TX 79601, USA

**Keywords:** brain tumor, autophagy, STAT3, drug repurposing, medulloblastoma, glioblastoma

## Abstract

Brain tumors are considered as one of the most aggressive and incurable forms of cancer. The majority of the patients with brain tumors have a median survival rate of 12%. Brain tumors are lethal despite the availability of advanced treatment options such as surgical removal, chemotherapy, and radiotherapy. In this study, we have evaluated the anti-cancer effects of pimozide, which is a neuroleptic drug used for the treatment of schizophrenia and chronic psychosis. Pimozide significantly reduced the proliferation of U-87MG, Daoy, GBM 28, and U-251MG brain cancer cell lines by inducing apoptosis with IC_50_ (Inhibitory concentration 50) ranging from 12 to 16 μM after 48 h of treatment. Our Western blotting analysis indicated that pimozide suppressed the phosphorylation of STAT3 at Tyr705 and Src at Tyr416, and it inhibited the expression of anti-apoptotic markers c-Myc, Mcl-1, and Bcl-2. Significant autophagy induction was observed with pimozide treatment. LC3B, Beclin-1, and ATG5 up-regulation along with autolysosome formation confirmed the induction of autophagy with pimozide treatment. Inhibiting autophagy using 3-methyladenine or LC3B siRNA significantly blocked the apoptosis-inducing effects of pimozide, suggesting that pimozide mediated its apoptotic effects by inducing autophagy. Oral administration of 25 mg/kg pimozide suppressed the intracranially implanted U-87MG tumor growth by 45% in athymic nude mice. The chronic administration of pimozide showed no general signs of toxicity, and the behavioral activity of the mice remained unchanged. Taken together, these results indicate that pimozide inhibits the growth of brain cancer by autophagy-mediated apoptosis.

## 1. Introduction

The brain is one of the most complex and delicate organs in the human body. Amongst all the malignant neurological conditions, brain cancer is the most life-threatening disease worldwide. Brain cancer accounts for 2% of all cancer types in the United States [1,2]. Brain tumors arise from different types of brain cells and the membranes associated with the brain. The conventional treatments for brain tumors include surgery, chemotherapy, and radiation therapy. The challenges associated with the treatment of brain tumors include the following: (1) recurrence of the brain tumor after surgery, (2) presence of the blood–brain barrier in the brain, (3) development of resistance to therapy over a prolonged treatment regime, and (4) heterogeneous nature of tumor cells [3]. Although several studies have provided intriguing insights regarding the mechanisms of brain tumor growth and therapy resistance, the survival rate for brain tumor patients remains < 1%, and it has been consistent over the past 30 years [4]. Hence, it is critical to develop novel treatment strategies and identify new treatment options for brain cancer management.

The dysregulation of oncogenic signaling pathways is a hallmark of cancer. Several studies have demonstrated the role of Janus Kinase-Signal Transducer and Activator of Transcription signaling (JAK-STAT signaling) signaling in various cancer models including brain tumors [5,6,7]. The JAK–STAT signaling pathway involves the canonical activation of JAK kinase or other growth factor receptor kinases [8]. The activation of JAK kinase mediates the activation of STAT3, which is an important transcription factor. STAT3 is a downstream effector and mediator of cytokine and growth factor signaling. Activated STAT3 dimerizes and translocates into the nucleus and regulates the transcription of several oncogenes including Mcl-1, Bcl-2, and c-Myc [9]. These oncogenes play a critical role in cell proliferation, homeostasis, cellular transformation, and cell cycle maintenance [10]. Recent studies have suggested STAT3 signaling to be directly associated with the progression of several tumors such as brain cancer, breast cancer, prostate cancer, and lung cancer [11]. STAT3 up-regulation has also been associated with the poor prognosis of patients [12]. The Bcl-2 family of proteins belongs to the anti-autophagy and anti-apoptotic group of genes. Autophagy is an evolutionarily conserved cellular homeostatic mechanism that mediates the lysosomal degradation of nonessential cellular components. Several extra or intracellular mechanisms can trigger autophagy. These mechanisms include stress, nutrient deprivation, DNA damage, and hypoxia, to name a few [13]. Autophagy involves the formation of a double membrane vesicle called the autophagosome. The fusion of autophagosomes with lysosomes generates autophagolysosomes that degrade the nonessential components by forming an acidic vesicle [14]. STAT3 has been reported to transcriptionally activate Bcl-2 expression. Bcl-2 forms a complex with Beclin 1 that leads to the inactivation of Beclin 1. Consequently, this complex formation promotes the inhibition of autophagy-mediated apoptosis [15,16,17].

Brain tumors, specifically glioblastoma multiforme, have been shown to exhibit low mRNA and protein levels of Beclin 1 [18]. Moreover, high Beclin 1 expression is considered to be in positive correlation with increased patient survival [19]. Several studies have highlighted the significance of STAT3 inhibition and its effect on the induction of autophagy [20]. Conventional therapeutic strategies involved in brain tumor treatment include the induction of apoptosis. However, resistance to apoptosis is a hallmark of tumorigenic progression [21]. Recently, arsenic trioxide has been shown to inhibit STAT3 activation, but it up-regulated autophagy in glioblastoma cells [22]. Hence, there is an emerging need for therapeutics with reduced toxicity and better efficacy toward brain tumors.

It has been documented that schizophrenic patients taking anti-psychotic medication have reduced incidence of cancer [14]. Anti-psychotic agents such as chloropromazine and thioridazine have been shown to induce autophagy-mediated cell death [23]; however, toxicities associated with these drugs have led to fatal outcomes clinically. Our previous studies have shown that penfluridol (an anti-psychotic drug) can suppress pancreatic tumor growth by inducing autophagy-mediated apoptosis [14,24].

**Figure 1:**
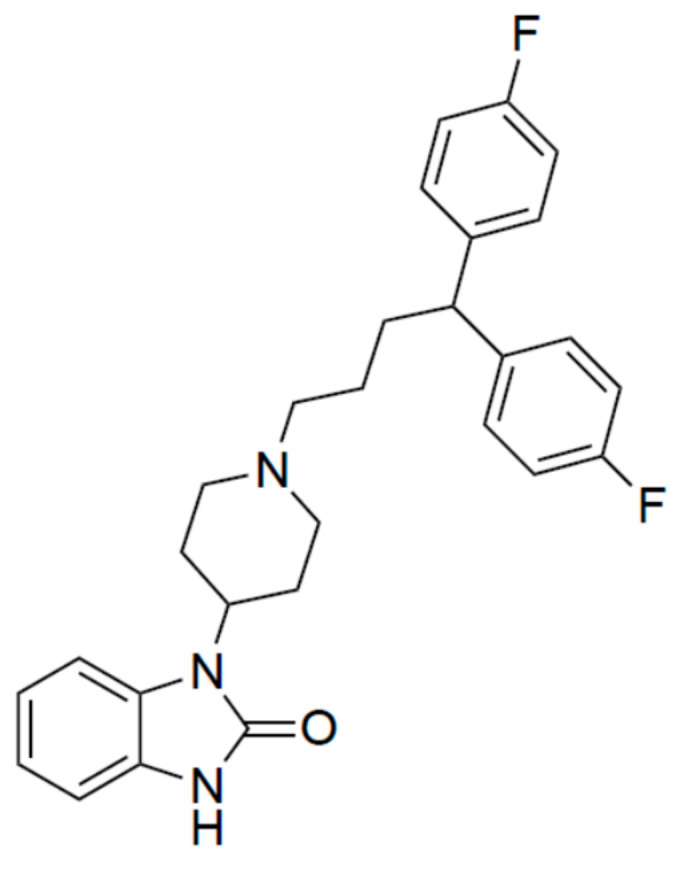
Structure of pimozide.

In the present study, we investigated the anti-cancer effects of pimozide (Figure 1) in brain cancer. We observed that pimozide significantly suppressed the growth of brain cancer cells by modulating STAT3-mediated autophagy signaling. The reduced expression of STAT3 with pimozide treatment resulted in increased autophagy, which in turn enhanced apoptosis in brain cancer cells. Moreover, the oral administration of pimozide suppressed the growth of brain tumors by inhibiting STAT3 and inducing autophagy-mediated apoptosis.

## 2. Material and Methods

### 2.1. Ethics Statement

All the animal experiments were conducted in accordance with the ethical standards and according to approved protocol by Institutional Animal Care and Use of Committee (IACUC), Texas Tech University Health Sciences Center.

### 2.2. Cell Culture

Human pediatric desmoplastic cerebellar medulloblastoma cell line Daoy and adult human brain cancer cell line U-87 MG were obtained from the American Type Culture Collection (ATCC), Manassas, VA, USA. We started working on U-87 MG cells 5 years ago when there was no question regarding its origin. Unfortunately, U-87 MG has been reported to have a different origin than the reported GBM tumors. However, it is still considered to be from tumors arising in the brain. The patient-derived glioblastoma cells GBM28 and U-251 MG used in this study were generously provided by Dr. Jann N Sarkariya, Mayo Clinic, Rochester, Minnesota. Daoy, U-251 MG, and GBM 28 cells were maintained in DMEM supplemented with 10% FBS and 1% PSN. U-87 MG cells were maintained in MEM media supplemented with 10% FBS and 1% PSN. All the cells used in this study were within twenty passages after receipt.

### 2.3. Cell Survival Assay

Brain tumor cells were plated at a density of ≈3000–4000 cells/well in 96-well plates and incubated overnight. Then, cells were treated with varying concentrations of pimozide (Sigma-Aldrich, St. Louis, MO, USA). After a desired treatment time point (24, 48 and 72 h), cells were fixed using ice cold 10% trichloroacetic acid followed by washing with water and staining with sulforhodamine B (SRB) dye for 2 h. Plates were washed with 1% solution of acetic acid and allowed to dry. SRB-stained cells were dissolved in 10 mM Tris-base solution, an optical density was measured using a plate reader (BioTek Instruments, Winooski, VT, USA) as described previously [3,25]

### 2.4. Clonogenic Assay

Briefly, 500 cells per well were plated in a six-well culture plate and incubated at 37 °C for 24 h. Then, plates were treated with 5, 10, 15, and 20 µM pimozide for 48 h. Media was replaced after 48 h, and the cells were incubated at 37 °C for 12-15 days. Once significant colonies were observed in the control group, the experiment was terminated, and colonies were fixed using ice-cold 10% trichloroacetic acid and staining with SRB dye. After ≈2 h of staining, the plates were washed with 1% solution of acetic acid and dried. The colonies from the control and treated group were quantified using Image J software.

### 2.5. AnnexinV/FITC Apoptosis Assay

AnnexinV/FITC apoptosis assay was performed using a commercially available kit and according to manufacturer’s instructions (BD Biosciences, San Jose, CA, USA). Approximately 0.2–0.3 × 10^6^ cells were plated in each well of a six-well plate and incubated overnight at 37 °C. Then, cells were treated with the increasing concentrations of pimozide for 48 h. Post 48 h of treatment, cells were trypsinized and washed twice with 1× PBS. An equal number of cells was suspended in 150 µL of binding buffer. Then, 4 µL Annexin-V FITC and 4 µL propidium iodide were added in every sample followed by incubation for 20 min in dark. Volume was adjusted to 400 µL by adding binding buffer. Samples were analyzed by flow cytometer (Accuri C6, Ann Arbor, MI, USA) after gently mixing the cells as described previously [3].

### 2.6. Western Blot Analysis

U-87MG, U-251MG, GBM 28, and Daoy cells were treated with 5, 10, 15, and 20 µM of pimozide for 48 h. Then, cells were collected and washed using 1X PBS solution twice. The whole cell lysates were prepared using 4% CHAPS in urea-tris buffer. Protein content in the lysates was estimated using Bradford reagent (Bio-rad, Irvine, CA, USA). Then, 25-40 µg of protein was subjected to SDS gel electrophoresis, and resolved proteins were transferred onto a PVDF membrane. The membranes were probed for primary antibody against p-STAT3 (Tyr705), STAT3, p-Src (Tyr416), MCl-1, BCl-2, Cleaved PARP, and Cleaved Caspase-3. β-Actin was used as a loading control (Cell Signaling and Technologies (Danvers, MA, USA)). The membranes were developed as described previously [26,27]

### 2.7. Acridine Orange Staining for Microscopy

Daoy cells were seeded in a six-well plate at a density of 0.2 × 10^6^. Cells were allowed to attach overnight, after which cells were treated with pimozide at a concentration of 10 µM for 48 h. After treatment, media was replaced with 1× PBS in each well. Then, 2-3 drops of NucBlue live cell stain (Life technologies, Eugene, OR, USA) were added to each well for 5 min. Acridine orange was added at a concentration of 0.4 µg/mL to each well, and images were taken using fluorescence microscope (Olympus, Center Valley, PA, USA)

### 2.8. Treatment with 3-Methyladenine

U-87 MG and Daoy cells were plated at a density of 0.3 × 10^6^ cells in a six-well plate. After overnight incubation, cells were pretreated with 5 mM 3-methyladenine (Sigma-Aldrich, St. Louis, MO) for 3 h. After pretreatment, cells were treated with 10 µM and 15 µM pimozide for 48 h and processed for Western blot analysis as previously described [26,27].

### 2.9. LC3B Silencing

U-87 MG and Daoy cells were transfected with LC3B siRNA (Cell Signaling Technologies, Danvers, MA, USA) using Lipofectamine RNAiMAX Transfection reagent (Thermo Fisher Scientific, Waltham, MA, USA). Cells were transfected with 100 nm LC3B siRNA or Scrambled control siRNA and 24 h post transfection, cells were treated with 10µM or 15µM pimozide for 48 h, as described by us previously [14]. After the treatment, cells were collected and processed using Western blot analysis.

### 2.10. shRNA Transfection

pSIH1-puro-STAT3 shRNA plasmid (plasmid #26596) was obtained from Addgene, and the plasmid DNA was isolated using a ZymoPURE^TM^ Plasmid Maxiprep Kit (catalog no: D4203) as described in the manufacturer’s protocol. Once the plasmid was isolated, it was quantified using Nano Drop 2000 spectrophotometer. Approximately 0.2 million Daoy cells were seeded in a six-well plate. The following day, cells were transfected with STAT3 shRNA using Xfect Transfection Reagent (Takara Bio, USA, lnc., Mountain View, CA, USA) in serum-free medium. With 5µg of STAT3 shRNA or control shRNA in serum-free medium, Daoy and U-251 MG cells were transfected as described in the manufacturer’s protocol and described by us previously [28]. Daoy and U251-MG cells were incubated overnight with shRNA, and approximately 12 h post transfection, the medium was replaced with suitable cell medium, and the cells were treated with 15 µM pimozide for 48 h. After 48 h of treatment, cells were collected and processed for Western blot analysis.

### 2.11. Intracranial Brain Tumor Implantation

Female athymic nude mice (4–6 weeks old) from Harlan Laboratories (Livermore, CA, USA) were used for intracranial injection, and the experiments were conducted in strict compliance with the Institutional Animal Care and Use Committee (IACUC). U-87 MG Luc cells were harvested, washed twice with sterile PBS, and resuspended in PBS at a density of 30 × 10^6^ cell per mL. A suspension of 5 µL containing 0.15 × 10^6^ cells was intracranially injected using a stereotaxic apparatus as described by us previously [3]. The growth of tumors was monitored using an IVIS imager. Once the tumors stabilized and exponential growth was observed, mice were randomized with eight mice in each group. The control group received the vehicle (water/PEG300/ethanol/2%acetic acid in 8:3:3:1 *v/v*), while the treated group received 25 mg/kg pimozide by oral gavage every day. The experiment was terminated at day 60 by euthanizing the mice using CO_2_ overdose, and each mice brain was aseptically dissected out and imaged for luminescence. A few brains from the control and treatment groups were snap frozen for Western blotting analysis. The mice weight and individual organ weight was measured to identify any general signs of toxicity with pimozide treatment.

### 2.12. Mice Behavioral Analysis

Mice from the control and treatment group were analyzed for general signs of toxicity and behavioral side effects due to pimozide treatment. After 60 days of treatment with pimozide, the toxicity and behavioral activity of mice was assessed using Versamax (AccuScan Instruments Inc., Columbus, OH, USA), as described by us previously [29].

## 3. Results

### 3.1. Pimozide Suppresses the Proliferation Of Brain Cancer Cells

**Figure 2:**
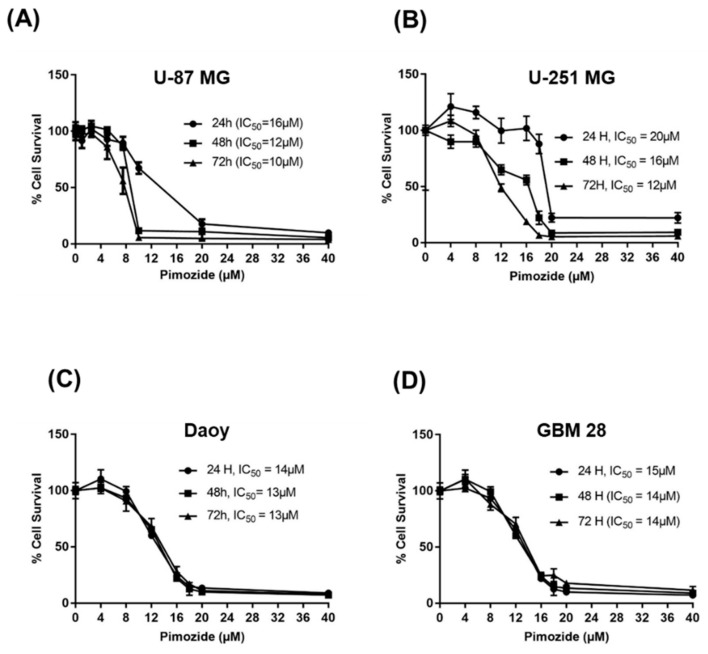
Pimozide suppresses the survival of brain tumor cells. (**A**) U-87 MG, (**B**) U-251 MG, (**C**) Daoy, and (**D**) GBM 28 cells were treated with different concentrations of pimozide for 24, 48, and 72 h. Cell survival was measured using Sulforhodamine B assay to estimate the IC_50_ values. The experiment was repeated three times with eight replicates in each experiment.

The treatment of U-87MG, U-251MG, Daoy, and GBM 28 cells with varying concentrations of pimozide displayed significant reduction in the survival of cells in a concentration and time-dependent manner (Figure 2A–D). The IC_50_ of pimozide was observed to be between 10 and 20 µM in U-87MG, Daoy, GBM-28, and U-251MG at 24, 48, and 72-h time points. These results indicate that pimozide has the ability to suppress the proliferation of brain cancer cells in a concentration and time-dependent manner.

### 3.2. Pimozide Inhibits the Colony Formation in Brain Cancer Cells

**Figure 3:**
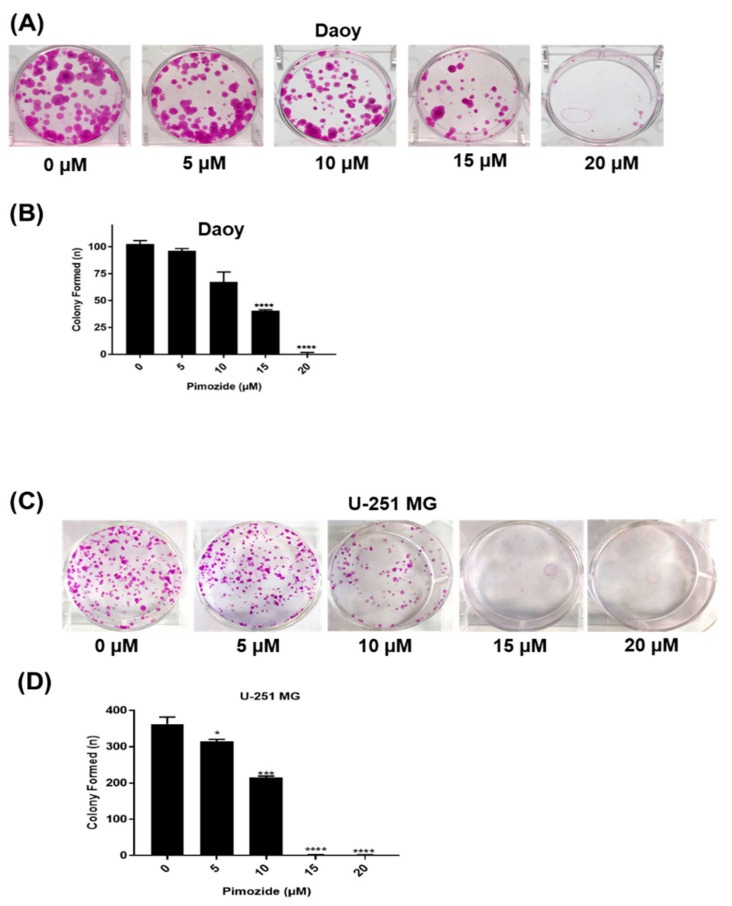
Pimozide inhibits the colony-forming ability of brain tumor cells. (**A**) Daoy cells were treated with sub-toxic and toxic concentrations of pimozide (5 µM, 10 µM, 15 µM, and 20 µM) for 48 h. The colony-inhibiting effects of pimozide were assessed by clonogenic assay. (**B**) Quantitative representation of the colonies formed in Daoy cells. The experiments contained two replicates in each experiment. Values are plotted as mean ± SD, and the statistical significance level was considered as *p* < 0.05 **** *p* ≤ 0.001) (**C**) U-251 MG cells were treated with sub-toxic and toxic concentrations of pimozide (5 µM, 10 µM, 15 µM, and 20 µM) for 48 h. The colony-inhibiting effects of pimozide were assessed by clonogenic assay. (**D**) Quantitative representation of the colonies formed in U-251 MG cells. The experiments contained two replicates in each experiment. Values are plotted as mean ± SD, and the statistical significance level was considered as *p* < 0.05 (* *p* ≤ 0.5, *** *p* ≤ 0.01, **** *p* ≤ 0.001).

The colony-forming ability of brain cancer cells after pimozide treatment was evaluated by clonogenic assay. Daoy and U251-MG cells were treated with sub-toxic concentrations of pimozide. Our observations indicate that 15 µM pimozide inhibited 70% of colony formation, whereas 20 µM of pimozide was able to completely inhibit the colony-forming ability of Daoy cells. However, 5 µM and 10 µM pimozide inhibited only 5–30% of colonies in Daoy cells (Figure 3A,B). Similarly, in U-251 MG glioblastoma cells, 10 µM pimozide treatment inhibited 60% of colonies, while 15 µM and 20 µM pimozide were able to completely inhibit the colony-forming ability of these cells. However, 5 µM pimozide was only able to inhibit 12% of colonies formed by U-251 MG cells (Figure 3C,D). These results indicate the anti-clonogenic activity of pimozide in brain cancer cells.

### 3.3. Induction of Apoptosis by Pimozide

**Figure 4:**
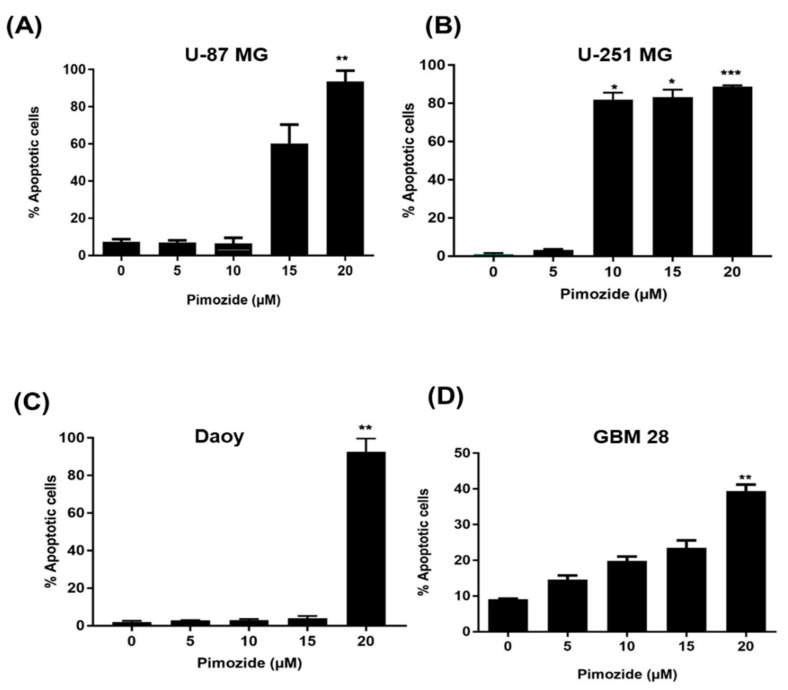
Pimozide induces apoptosis in brain tumor cells in a concentration-dependent manner. Approximately 0.2 × 10^6^ of (**A**) U-87 MG, (**B**) U-251 MG, (**C**) Daoy, and (**D**) GBM 28 cells were plated in six-well plates, treated with 5, 10, 15, and 20 µM pimozide for 48 h and processed for AnnexinV/FITC apoptosis assay using a BD FACSverse flow cytometer. Values were plotted as mean ± SD. The experiment was repeated three times. * Statistically significant at *p* ≤ 0.05 when compared with control (* *p* ≤ 0.5, ** *p*≤ 0.1, *** *p* ≤ 0.01).

**Figure 5:**
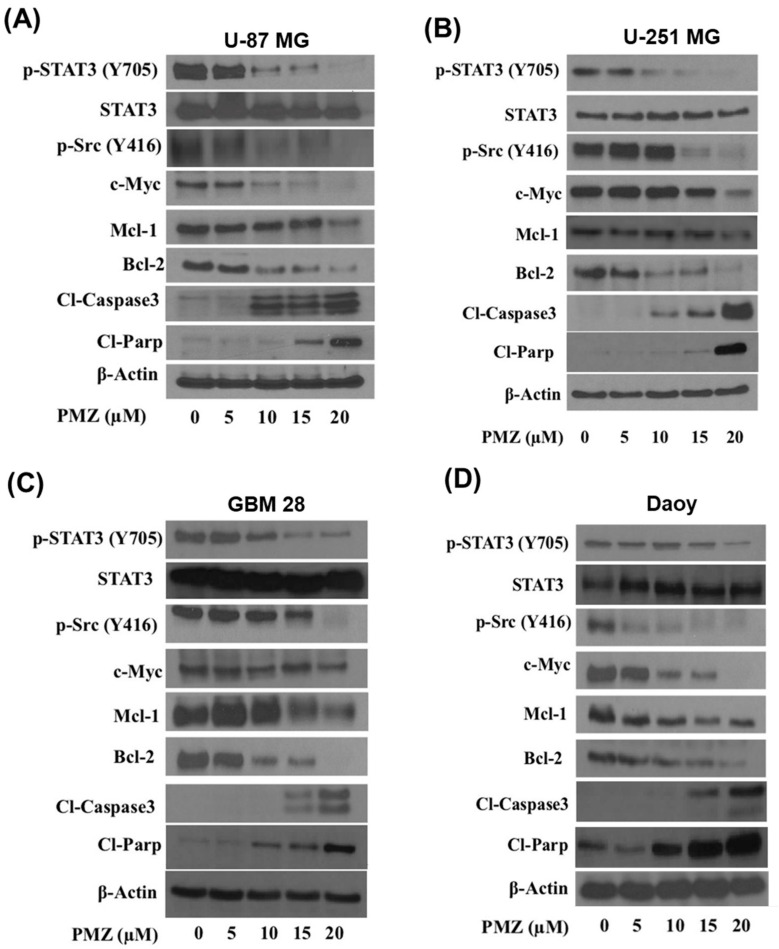
Pimozide inhibits STAT3 signaling. Approximately, 0.7 million of (**A**) U-87 MG, (**B**) U-251 MG, (**C**) GBM28, and (**D**) Daoy cells were treated with 5, 10, 15, and 20 µM pimozide for 48 h. Representative blots showing a concentration-dependent effect of pimozide on pSTAT3 (Tyr705), STAT3, p-SRC (Tyr416), c-Myc, Mcl-1, Bcl-2, cleaved caspase 3, and cleaved PARP. β-actin was used as a loading control. Figures shown are the representative blots of at least three independent experiments.

The mode of cell death caused by pimozide was determined by Annexin V/FITC assay in U-87MG, U-251MG, Daoy, and GBM-28 brain cancer cells. As shown in Figure 4, treatment with pimozide for 48 h resulted in significant apoptosis in all brain cancer cells. Treatment with 5 µM and 10 µM pimozide had minimal apoptotic effects on U-87 MG and Daoy cell lines (Figure 4A–D); however, similar concentrations of pimozide induced apoptosis in GBM-28 and U-251MG cells. The percentage of apoptotic cells with 10 µM pimozide ranged between 5% and 75%, whereas 15µM pimozide induced 10–80% apoptosis in U-87 MG, U-251MG, and GBM 28-cells. The percentage of apoptotic cells with 20 µM pimozide treatment ranged from 30% to 90% in all the brain cancer cell lines tested, with U-87MG and Daoy being the most sensitive cell lines at this concentration (Figure 4A–D). The induction of apoptosis by pimozide treatment in these cell lines was further confirmed by increase in the cleavage of caspase 3 and PARP as assessed by Western blotting (Figure 5A–D).

### 3.4. Pimozide Inhibits STAT3 Signaling

In order to delineate the mechanism by which pimozide mediates its anti-cancer effects, brain cancer cell lines, U-87MG, U-251MG, Daoy, and GBM-28 cells were treated with 0, 5, 10, 15, and 20 µM pimozide for 48 h and evaluated by Western blotting. Our results showed that pimozide treatment reduced the phosphorylation of STAT3 at Tyr705 in a concentration-dependent manner. However, the protein level of STAT3 remained unchanged. It has been reported that STAT3 is transcriptionally activated by non-receptor tyrosine kinases such as Src kinase [21]. Our results indicated that pimozide treatment reduced the phosphorylation of Src kinase at Tyr416. The hyperactivation of STAT3 in brain cancer cells has been strongly attributed to the evasion of apoptosis. The transcriptional activation of anti-apoptotic proteins such as Bcl-2 and Mcl-1 by STAT3 has been documented in several studies [30,31,32]. Interestingly, we observed that the treatment of brain cancer cells with pimozide resulted in a reduced expression of anti-apoptotic proteins Mcl-1 and Bcl-2 in U-87 MG, U-251 MG, GBM 28, and Daoy cell lines (Figure 5A–D). Moreover, pimozide increased the cleavage of caspase 3 and PARP, which is an indication of apoptosis (Figure 5A–D). These results suggest that the anti-proliferative and apoptotic effects of pimozide were mediated by inhibiting STAT3 activation and its downstream targets Mcl-1 and Bcl-2.

### 3.5. Pimozide Induces Autophagy in Brain Cancer Cells

**Figure 6:**
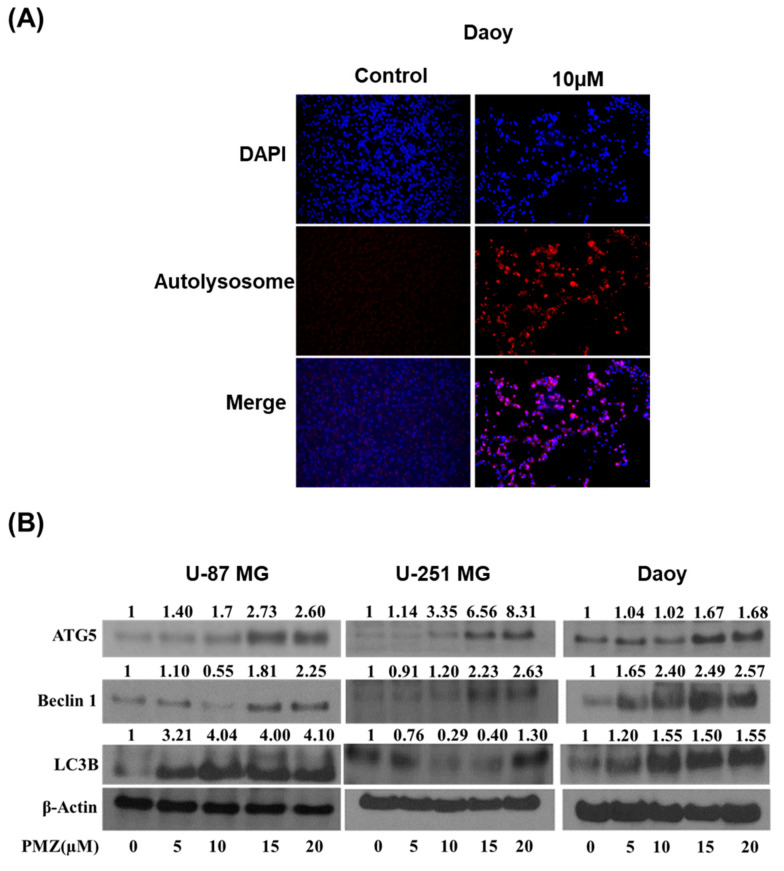
Production of autolysosome by pimozide treatment. (**A**) Approximately 0.2 million Daoy cells were plated in a six-well plate. Daoy cells were treated with 10 µM of pimozide. After 48 h of pimozide treatment, cells were stained with NucBlue followed by staining with 0.4 µg/mL acridine orange. Images were taken using a florescence microscope. Blue florescence represents DAPI, whereas red represents the formation of autophagolysosomes. (**B**) Approximately 0.7 million U-87 MG, Daoy, and U-251 MG cells were treated with different concentrations of pimozide for 48 h. Representative blots show the concentration-dependent effect of pimozide on ATG5, Beclin1, LC3B, cleaved caspase 3, and cleaved PARP expression. β-actin was used as a loading control. Figures shown are the representative blots of at least three independent experiments.

We observed a significant induction of apoptosis with pimozide treatment in brain cancer cells. Our observations suggest that the apoptotic effects of pimozide were mediated through STAT3 inhibition. STAT3 transcriptionally regulates several genes that induce autophagy [13,33,34]. In order to investigate the underlying mechanism of apoptosis and the effect of pimozide on autophagy-mediated apoptosis, acridine orange assay was performed. Acridine orange is a cell-permeable green fluorophore. It can get protonated inside an acidic vesicular organelle (AVO). Acridine orange fluoresces with a red color when its concentration increases inside an AVO [35]. Interestingly, we observed that pimozide treatment induced autophagy in a concentration-dependent manner in Daoy cells at the 48 h time point. A significant increase in red staining indicated the production of AVOs (Figure 6A). Furthermore, our Western blot analysis showed an up-regulation of LC3B, which is a marker for the formation of autophagic vesicles, Beclin-1 and ATG5 in U-87 MG, U-251 MG, and Daoy brain cancer cells (Figure 6B). These results prove the pro-autophagy effects of pimozide in brain cancer cells.

### 3.6. Silencing LC3B and Inhibiting Autophagy Abrogates the Apoptotic Effects of Pimozide

**Figure 7:**
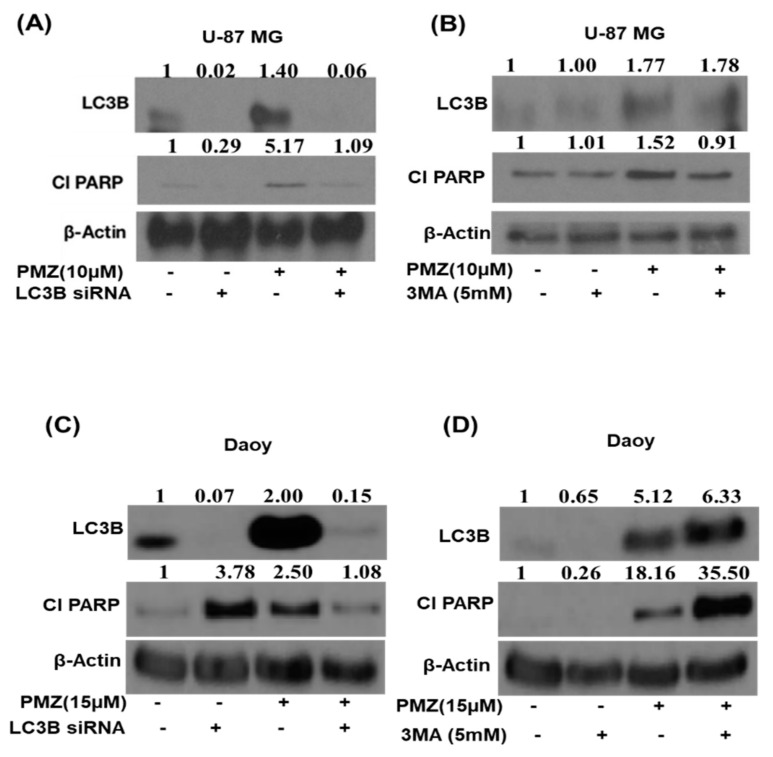
Pimozide induces autophagy-mediated apoptosis. (**A**,**C**) Approximately 0.2 million U-87 MG and Daoy cells were treated with LC3B siRNA followed with the treatment of 10 µM and 15 µM pimozide for 48h. Levels of LC3B and cleaved PARP were evaluated by Western blotting. β-actin was used as a loading control. (**B**,**D**) U-87 MG and Daoy cells were pretreated with 5 mM 3-methyladenine for 3 h. Following the treatment of cells with an autophagy inhibitor, cells were treated with 10 µM and 15 µM pimozide for 48 h. The expression of LC3B and cleaved PARP was evaluated using Western blot analysis. β-actin was used as a loading control. The figure is a representation of at least three independent experiments.

To validate the role of autophagy in pimozide-mediated apoptosis, we genetically knocked down LC3B expression in U-87 MG and Daoy cells using LC3B siRNA before treatment with 10 µM or 15 µM pimozide for 48 h (Figure 7A–C). Our results demonstrated that pimozide treatment reduced the cleavage of PARP, which is an indicator of apoptosis in LC3B siRNA transfected cells. These results confirmed that pimozide treatment induced autophagy-mediated apoptosis. In another experiment, U-87 MG and Daoy cells were pretreated for 3 h with a known autophagy inhibitor 3-methyladenine (3-MA) followed by treatment with 10 µM and 15 µM pimozide for 48 h. Blocking autophagy resulted in reduced apoptosis as indicated by a decrease in the cleavage of PARP by pimozide treatment in U-87 MG cells (Figure 7B). These results indicate the regulation of apoptosis by autophagy in our model.

### 3.7. STAT3 Knockdown Potentiates the Autophagic Effects of Pimozide

**Figure 8:**
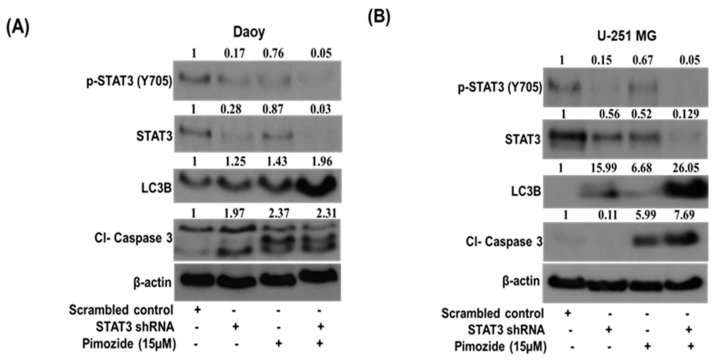
Pimozide suppresses the growth of brain tumor cells by inducing STAT3-mediated autophagy. (**A**,**B**) Approximately 0.2 million Daoy and U-251 MG cells were transfected with 5 µg of STAT3 shRNA followed with the treatment of 15 µM pimozide for 48 h. STAT3 inhibition, the induction of autophagy marker LC3B, and the level of apoptosis induction were evaluated by Western blotting analysis. β-actin was used as a loading control. The figure is a representation of at least three independent experiments.

STAT3 knockdown was performed in Daoy and U251-MG cells using shRNA to validate the STAT3-mediated autophagy effects of pimozide. As per Western blotting analysis, we observed approximately 72% inhibition of STAT3 in Daoy cells and approximately 44% inhibition in U-251 MG cells post STAT3 shRNA transfection. Our results indicated that knocking down STAT3 using shRNA does not significantly induce autophagy on its own in brain cancer cells. However, we observed an increase in autophagy as well as autophagy-mediated apoptosis with the treatment of STAT3 shRNA-transfected cells with 15 µM pimozide (Figure 8A,B). These results indicate that pimozide induces apoptosis in brain cancer cells by inducing autophagy

### 3.8. Pimozide Suppresses the Growth of Intracranially Implanted Brain Cancer Cells

**Figure 9:**
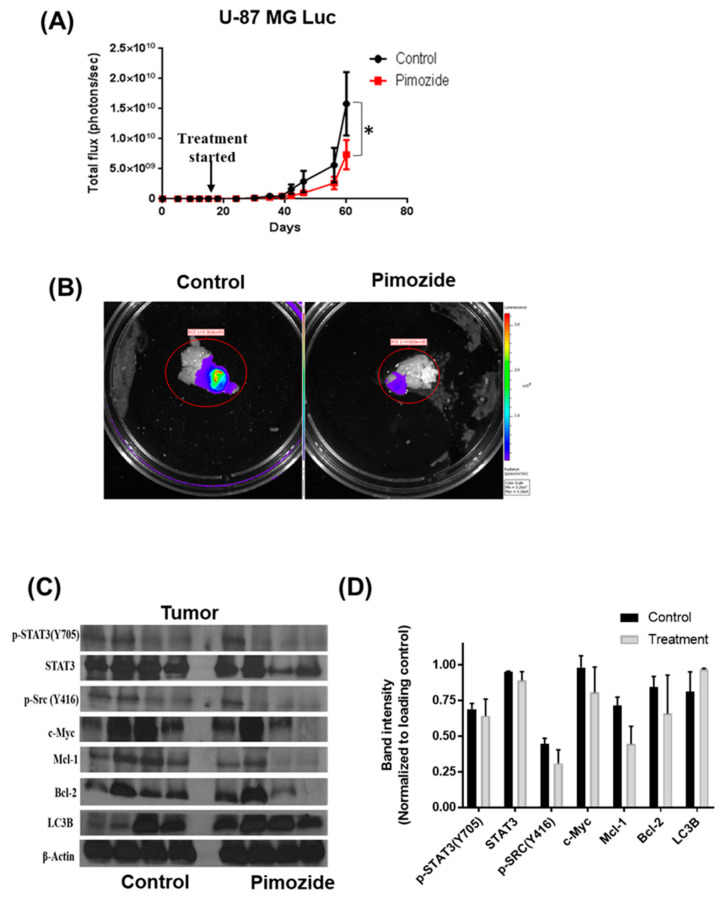
Pimozide suppresses the growth of intracranially implanted brain tumor cells. (**A**) About 0.15 × 10^6^ U-87MG luc cells were implanted intracranially in the brain of 4–6-week-old athymic nude mice. Mice were treated with 25 mg/kg pimozide starting day 14 after tumor cells were implanted until day 60. Representative luminescence (photons/second) was measured about thrice a week and plotted against days. The statistical significance level was considered as * *P* ≤ 0.05. (**B**) Representative mouse brain from the control and pimozide-treated groups after terminating the experiment. (**C**) After terminating the experiment, brains with tumors were removed aseptically, lysed, and analyzed for p-STAT3, STAT3, p-src, Mcl-1, Bcl-2, and LC3B. (**D**) Graphical representation of the protein expression in control and treatment groups when normalized with its respective β-actin.

To evaluate the ability of pimozide in inhibiting the growth of brain tumor cells in vivo, U-87 MG luc cells were injected intracranially into the brain of mice using stereotaxic apparatus. Once the tumor cells started growing, mice were randomized into two groups. The treatment group received 25 mg/kg pimozide every day by oral gavage, while the other group served as control and received vehicle only. The growth of tumor in mice was evaluated by using an IVIS imager. Our results showed that pimozide treatment suppressed the growth of intracranially implanted U-87 MG tumors by 45% (Figure 9A). We also analyzed luminescence in the isolated brains from both the control and treatment group. The average luminescence in the treated group was 70% less compared to the control group (Figure 9B). To further validate the in vitro results, whole brain lysates of the control and treatment groups were analyzed by Western blotting. In agreement with our in vitro results, the pimozide-treated group of mice showed a reduction in the activation of STAT3 along with a reduced expression of anti-apoptotic proteins such as Mcl-1 and Bcl-2. Furthermore, pimozide-treated mice showed an increased expression of autophagy marker LC3B (Figure 9C). As an indicator of apoptosis, the increased expression of cleaved caspase 3 and PARP was observed with pimozide treatment. Taken together, these results suggest that reduced tumor growth in the brain by pimozide was due to autophagy-mediated apoptosis.

### 3.9. Pimozide Treatment Display No Major Side Effects in Mice

**Figure 10:**
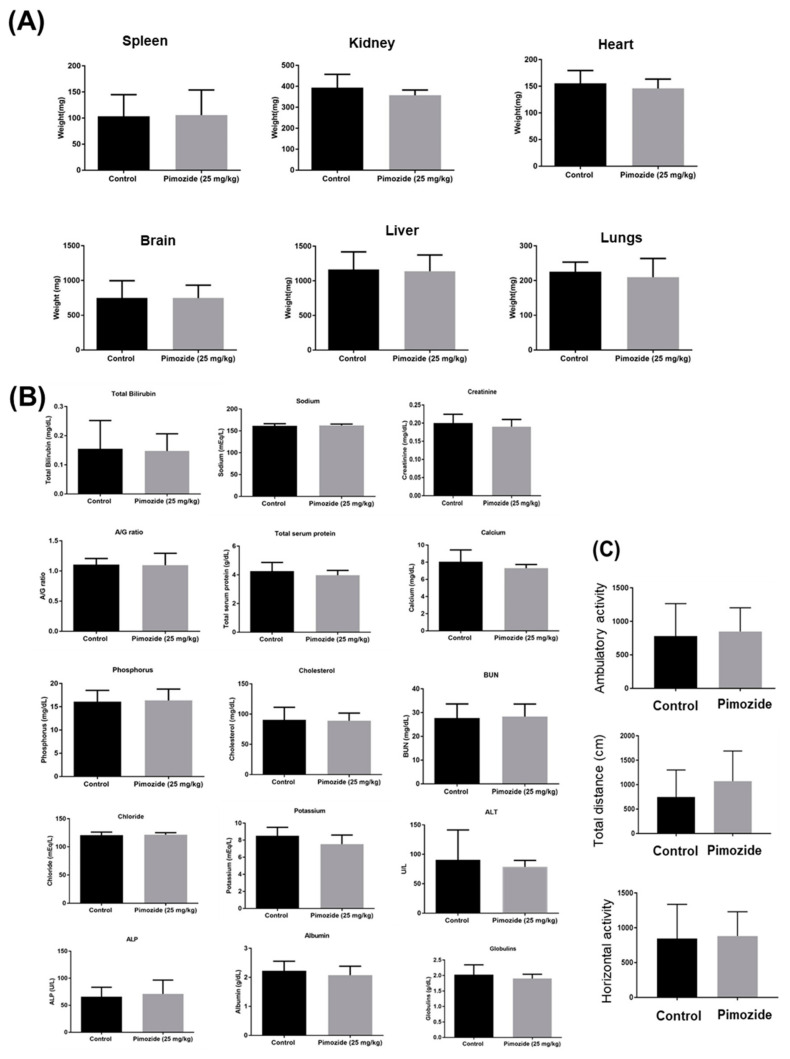
Safety profile study in mice. About 0.15 million U-87 MG cells were implanted intracranially in the brain of 4–6-week-old female athymic nude mice. Once tumors achieved a minimum size, 25 mg/kg pimozide by oral route was administered every day to mice. (**A**) Mice were observed for general signs of toxicity, and their organ weight was measured in comparison with the control group. (**B**) After 60 days, mice were sacrificed, and plasma was sent for analysis to Texas Veterinary Medical Diagnostic Laboratory System, Amarillo. A comparative analysis of liver enzymes, metabolic enzymes, and ions in the plasma of the control and treatment groups was performed. (**C**) Before the termination of the experiment, mice were analyzed for any behavioral side effect due to pimozide treatment using Versmax.

To evaluate the general signs of toxicity, mice weight and organ weight were recorded. In our analysis, we observed no significant difference in the average organ weight of the control and pimozide-treated mice (Figure 10A). Pimozide treatment did not alter kidney or liver functions nor blood chemistry in mice (Figure 10B). The long-term behavioral side effects such as locomotor activity were evaluated in both the control and pimozide-treated groups using Versamax. Our observations indicated that there was no significant difference in the locomotor activities such as ambulatory activity, total distance, or horizontal activity of the mice (Figure 10C). These results indicate that the chronic administration of pimozide for treating brain tumors may not have any critical side effects in mice. However, further detailed studies are required to validate the toxicity profile of pimozide.

## 4. Discussion

Glioblastoma is the most aggressive and incurable form of brain cancer in adults, whereas medulloblastoma is an aggressive form of brain cancer in pediatric patients [2,36]. In our study, we evaluated the anti-cancer effects of pimozide, an anti-psychotic drug in five different cell lines from glioblastoma and medulloblastoma, and patient-derived tumor cells [37,38]. Pimzoide belongs to the diphenylbutylpiperidine class of anti-psychotic drugs. It is prescribed as an oral preparation for schizophrenia, chronic psychosis, Tourette’s syndrome, and resistant tics [39]. Our current study demonstrates the anti-cancer effects of pimozide in vitro and in vivo against brain tumors.

Currently approved chemotherapeutic drugs display higher resistance and an enhanced chance of tumor recurrence. Temozolomide (TMZ) is one of the most commonly used drugs for the treatment of glioblastoma and medulloblastoma [40,41]. Temozolomide is an alkylating agent that mediates its effects by activating p53 and therefore inducing apoptosis in cancer cells. The activation of O6-methylguanine-methyltransferase (MGMT), a DNA repair enzyme in the cancer cells, impairs the activity of TMZ [42]. One of the reasons of increased resistance to TMZ could be the minimal cytotoxic effects of TMZ. The IC_50_ of TMZ in U-87 MG glioblastoma cells is approximately 100µM at the 144-h time point [43]. Therefore, identifying a compound with enhanced cytotoxic effects, which can potentiate the anti-cancer effects of TMZ, is imperative. Epidemiological studies have indicated that people with schizophrenia are less likely to develop glioma [44]. We observed that pimozide has the ability to inhibit cell proliferation and the colony-forming ability of various brain cancer cells. Pimozide exhibited a concentration and time-dependent growth-suppressive effect in all the tested cell lines, and the IC_50_ ranged from 12 to 16µM after 48 h of treatment. Additionally, we observed significant apoptosis with pimozide treatment. Several studies have demonstrated that glioblastoma and medulloblastoma patients express higher levels of STAT3 as compared to healthy individuals [45,46]. STAT3 stimulates oncogenesis by promoting cell proliferation, inhibiting apoptosis, suppressing anti-tumor immune response, and dysregulating the cell cycle, survival, and senescence. Phosphorylated (activated) STAT3 translocates to the nucleus and promotes the transcription of downstream genes such as Bcl-2, Mcl-1, and c-Myc [47,48]. Targeting STAT3 has shown to inhibit cell proliferation and induce apoptosis [49,50]. To the best of our knowledge, no FDA-approved drug has been able to specifically target STAT3 activation [51,52]. Our results indicated that pimozide treatment suppressed the activation of Src kinase, which is a non-receptor tyrosine kinase upstream of STAT3. Furthermore, pimozide treatment reduced the phosphorylation of STAT3 at the Tyr705 site in a concentration-dependent manner. The downstream effectors of STAT3 such as Mcl-1, Bcl-2, and c-Myc were also reduced by pimozide treatment.

Several scientific studies have established the role of STAT3 in autophagy [13,53,54,55]. Autophagy plays a significant role in regulating apoptosis [56,57]. Our previous studies have shown autophagy-mediated apoptosis by penfluridol treatment in GBM [14]. Herein, we evaluated the role of pimozide as an autophagy modulator. From our results, we inferred that pimozide treatment resulted in a concentration-dependent increase in the production of acidic autophagic vesicle formation indicating autophagy. In addition, pimozide treatment caused a concentration-dependent increase in the expression of autophagy markers such as LC3B, ATG5, and Beclin 1.

Bcl-2 is an apoptosis-inhibiting gene that is regulated by STAT3. Several studies have mentioned that Bcl-2 can inhibit autophagy by repressing the expression of Beclin-1. Thus, Bcl-2 inhibition can result in increased autophagy [58,59,60]. Our results showed that pimozide reduced the expression of Bcl-2 and enhanced autophagy. In order to establish the mechanism of autophagy-mediated apoptotic effects of pimozide, LC3B siRNA and 3-methyladenine (well-known autophagy inhibitor) were used. Blocking autophagy using a pharmacological inhibitor 3MA or siRNA exhibited reduced apoptosis by pimzoide treatment in U-87 MG cells. The treatment of Daoy medulloblastoma cells with LC3B siRNA reduced the apoptotic effects of PMZ; however, interestingly, the treatment of Daoy cells with the inhibitor 3MA slightly increased the apoptotic effects of PMZ. The increased apoptosis in the combined treatment of 3MA with PMZ can be attributed to the off-target effects of the inhibitor. Nonetheless, in general, our data are in agreement with the published studies demonstrating that induced autophagy causes cancer growth suppression [61]. Our results showed that inhibiting STAT3 induces autophagy, which in turn increases the apoptosis in brain cancer cells. In order to establish the role of STAT3 in inducing autophagy, STAT3 shRNA was used. Inhibiting STAT3 using shRNA potentiated the effects of pimozide by inducing autophagy and thereby increasing apoptosis in Daoy medulloblastoma cells. These observations are supported by several published studies that have shown the significant role of STAT3 in inducing autophagy and autophagy-mediated apoptosis [62,63].

Moreover, pimozide inhibited the growth of intracranially implanted tumor by 45% in mice. The behavioral activity remained unchanged in mice after the chronic administration of pimozide by the oral route. The weight of the mice and organ weight also did not change throughout the course of study. However, side effects associated with the long-term administration of pimozide cannot be ruled out. The tumors obtained from pimozide-treated mice displayed increased autophagy and apoptosis.

Taken together, these data suggest that pimozide has the ability to inhibit the growth of brain cancer in vitro and in vivo by inhibiting STAT3, leading to induced autophagy-mediated apoptosis. Overall, our study established autophagy-mediated anti-tumor effects of pimozide in brain cancer.
